# Nurses’ experiences with cardiovascular risk communication and lifestyle counselling during preventive dialogues in primary care: a qualitative interview study

**DOI:** 10.1186/s12875-026-03401-7

**Published:** 2026-06-04

**Authors:** Anna Sjöström, Åsa Hörnsten, Patrik Wennberg, Thorbjörn Lundberg, Albin Dahlin Almevall

**Affiliations:** 1 Department of Nursing, Umeå University, Umeå, Sweden; 2 Department of Public Health and Clinical Medicine, Umeå University, Umeå, Sweden; 3 Department of Health and Medical Care, Luleå, Region Norrbotten Sweden

**Keywords:** Cardiovascular disease, Primary prevention, Risk communication, Nursing, Person-centred care, Motivational interviewing, Behaviour change, Visualisation, Qualitative research

## Abstract

**Background:**

Cardiovascular disease (CVD) is the leading cause of morbidity and mortality globally and in Sweden. Effective primary prevention requires not only clinical risk assessment but also person-centred communication to promote a healthy lifestyle. Nurses play a key role in structured preventive dialogues, such as those in the Västerbotten Intervention Programme (VIP), one of the world’s largest prevention programmes. However, little is known about how nurses experience and navigate risk communication in real-world primary care. This study aimed to explore nurses’ experiences with cardiovascular risk communication and lifestyle counselling during preventive dialogues in primary care.

**Methods:**

Semistructured interviews were conducted with 11 nurses delivering VIP health dialogues across primary care centres in Västerbotten County between December 2024 and April 2025. The interviews were audio-recorded and transcribed verbatim. Data were analysed via qualitative content analysis, identifying meaning units, codes, categories, and an overarching theme. Reporting followed COREQ.

**Results:**

The analysis generated two main categories. The first, *Meeting the person professionally while taking a step back*, captured how nurses described exploring each patient’s unique situation, needs, motivations, and attitudes. The second, *Continuously adapting approaches in self-management support*, captured nurses’ accounts of using flexible strategies, such as gradual goal setting, visual tools, and practical daily habit suggestions, to support patients in understanding and working with lifestyle change. Together, these categories formed the overarching theme *Shifting between leading and following*, illustrating how nurses experienced balancing individualised adaptation with professional guidance while navigating structural and contextual challenges.

**Conclusions:**

Nurses described translating cardiovascular risk into person-centred, relational dialogue by flexibly combining structure, responsiveness, and motivational strategies. Understanding preventive dialogues as interactive, person-centred processes, supported by the sensitive use of visualisation tools, may inform the continued development of cardiovascular prevention in primary care and warrants further evaluation.

**Supplementary Information:**

The online version contains supplementary material available at 10.1186/s12875-026-03401-7.

## Background

Cardiovascular disease (CVD) remains the leading cause of death globally and in Sweden, accounting for approximately one-third of all deaths [42, 55]. Atherosclerosis underlies most cardiovascular events, such as myocardial infarction and stroke, and often progresses silently until advanced stages [1]. More than half of incident CVD cases and one-fifth of all-cause deaths are attributable to five modifiable risk factors: high body mass index, elevated blood pressure, high LDL cholesterol, tobacco use, and diabetes [17]. Key preventive behaviours, including a healthy diet, regular physical activity, smoking cessation, and weight management, substantially reduce cardiovascular risk [54]. Effective prevention therefore requires not only accurate risk assessment but also the translation of evidence into meaningful patient support, work in which primary care nurses play a central role. Nurses constitute the largest professional group involved in cardiovascular risk management and contribute through structured consultations that combine health education, motivational support, and personalised risk communication [20, 49]. Understanding how nurses experience and carry out this work in everyday primary care practice is therefore important.

Despite widespread access to health information, adherence to both lifestyle recommendations and preventive pharmacotherapy remains low [5, 22, 30]. Risk communication, defined as the open, two-way exchange of information and opinions about risk between health professionals and individuals, aims to enhance understanding, risk perception, and informed decision-making [2]. International research on lifestyle risk communication in primary care suggests that such communication is inherently relational, context-dependent, and shaped by patients’ readiness, motivation, and life circumstances [24]. Studies focusing on primary care nurses’ communication practices indicate that discussions about lifestyle risk are embedded in ongoing nurse–patient relationships and unfold as interactional processes, where rapport, trust, and sensitivity to patients’ perspectives are central [24, 25]. These studies also indicate that nurses employ a range of person-centred communication strategies, while at times using more directive or fear-based approaches when conveying risk [24].

Contemporary frameworks for lifestyle prevention emphasise autonomy-supportive dialogue, shared decision-making, and motivational approaches tailored to individuals’ values, preferences, and life circumstances [14, 39]. Long-term prevention is understood to depend not only on pharmacological treatment [29] but also on person-centred approaches that actively engage individuals in their own care [54]. In contrast, observational and review-based research from primary care suggests that lifestyle counselling in routine practice is often delivered in relatively general terms and that recommended communication approaches are applied inconsistently across consultations and professionals [43, 44]. Studies examining nurses’ experiences further highlight barriers related to time constraints, organisational conditions, and perceived patient motivation [4, 7, 26, 57]. However, this body of research has largely focused on nurses working in mixed inpatient and outpatient settings or on general counselling activities, rather than on nurses with a defined, preventive role within structured primary care programmes. Together, these findings point to a gap between recommended approaches to lifestyle counselling and the realities of everyday primary care practice. Lifestyle risk communication has therefore been conceptualised as a complex and dynamic process that requires both patient engagement and skilled professional support [35], yet important questions remain regarding how nurses experience, adapt, and enact such communication in routine preventive practice.

Although preventive efforts often focus on individuals with established risk factors such as hypertension, diabetes, or hyperlipidaemia, most CVD cases arise within the much larger group at low to moderate risk, underscoring the importance of population-based prevention strategies [16, 47]. In northern Sweden, such strategies have included structured, nurse-led preventive health dialogues in primary care as part of the Västerbotten Intervention Programme (VIP) [45]. While these programmes have demonstrated favourable population-level outcomes [10], translating communicated risk into sustained lifestyle change at the individual level remains a key challenge.

Within the specific context of preventive health dialogues in primary care, one qualitative study has shown that nurses navigate a balance between providing professional guidance and respecting patients’ autonomy, perspectives, and readiness for change [23]. As both primary care practice and preventive communication strategies have continued to evolve since that study was conducted, updated qualitative knowledge is needed to understand how nurses currently experience cardiovascular risk communication and lifestyle counselling in routine preventive practice. The aim of this study was therefore to explore nurses’ experiences with cardiovascular risk communication and lifestyle counselling during preventive health dialogues in primary care.

## Methods

A qualitative interview design with an inductive content analysis approach was used. This study is reported in accordance with the Consolidated Criteria for Reporting Qualitative Research (COREQ) checklist ([52], see Supplementary File).

### Study context

This study was conducted within the Västerbotten Intervention Programme (VIP), a long-standing, population-based cardiovascular disease prevention programme implemented in primary care in Västerbotten County, northern Sweden. The programme was initiated in 1990 in response to a high regional burden of cardiovascular disease and invites residents turning 40, 50, and 60 years of age to attend a health examination followed by a structured preventive health dialogue with a nurse [45].

Within the VIP, the preventive health dialogue is a central component and is primarily conducted by registered nurses, most commonly with postgraduate specialist training in primary care. The dialogue integrates clinical measurements, including blood pressure, blood lipids, and glucose levels, with self-reported lifestyle information. The clinical measurements are typically communicated as absolute values (e.g., blood pressure and cholesterol levels) and discussed in relation to cardiovascular risk and the potential benefits of lifestyle change, rather than being expressed as formal quantitative risk estimates such as percentages or calculated future risk. A visual pedagogical tool, commonly referred to as the “star profile”, is used to combine this information and support discussion about cardiovascular risk and lifestyle-related behaviours. Individuals identified as having elevated risk are referred for further medical assessment or treatment in accordance with regional guidelines. The preventive health dialogue is typically allocated approximately 60 min.

Nurses conducting VIP health dialogues complete a regionally approved training programme in the VIP model and in motivational interviewing (MI), aiming to support the use of structured, theory-based communication strategies within a person-centred, health-promoting approach [45, 48]. They also regularly participate in continuing professional education to stay updated on current evidence in the prevention of cardiovascular disease and type 2 diabetes [48]. Motivational interviewing (MI) is a collaborative, goal-oriented style of communication that seeks to strengthen individuals’ motivation and commitment to change by eliciting and exploring their own reasons for change within an atmosphere of acceptance and compassion. In practice, this involves the use of open-ended questions, reflective listening, and affirmation of the individual’s perspectives and strengths, as well as a structured conversational process in which the clinician engages with the individual, jointly identifies a focus for the dialogue, and supports exploration of motivations and possibilities for change [41].

The VIPVIZA trial (Västerbotten Intervention Programme – VIsualiZAtion of asymptomatic atherosclerotic disease) is a pragmatic, open-label randomised controlled trial conducted within the VIP framework. The trial investigates the use of carotid ultrasound to visualise subclinical atherosclerosis as a means of supporting cardiovascular risk communication within preventive health dialogues [46]. Building on this work, the present study forms part of the ongoing PRIMVIZA project (PRIMary Care Prevention of Cardiovascular Disease using VIsualiZAtion), which explores how ultrasound-based visualisation of subclinical atherosclerosis can be integrated into VIP health dialogues in routine primary care. Within PRIMVIZA, carotid ultrasound examinations are performed by trained nurse assistants using a digital examination and decision support system. The resulting visualisations are presented and discussed by the nurse during the preventive health dialogue [53].

Although the interviews for the present article were conducted within the context of the PRIMVIZA project, the present analysis focuses on nurses’ general experiences of cardiovascular risk communication and lifestyle counselling during VIP preventive health dialogues. Ultrasound-based visualisation is therefore not examined as a specific analytic focus in this study but constitutes part of the broader programme context.

### Setting and recruitment

In Swedish primary care, primary care nurses often serve as the first point of contact for individuals seeking medical advice and services. They commonly work with a high degree of professional autonomy and are expected to possess broad clinical competence, including the ability to assess, identify, and manage a wide range of health conditions in accordance with evidence-based practice [50]. Their work typically includes outpatient clinical activities such as health assessments, blood pressure measurements, wound care, and injections, as well as nurse-led clinics for patients with chronic conditions such as diabetes, asthma, or chronic obstructive pulmonary disease. In addition to clinical tasks, primary care nurses play a key role in health promotion, with an emphasis on patient empowerment and partnership, including shared decision-making in care and treatment [50].

Nurses who had been involved in VIP health dialogues for at least six months were purposively recruited for the study. Recruitment was facilitated through contact with unit managers at participating primary care centres, who provided information about the study and forwarded contact details for eligible nurses. Sixteen nurses who met the inclusion criteria were contacted and invited to participate. Of these, eleven agreed to participate, while five did not respond to the invitation. No reasons for non-participation were recorded. A total of 11 nurses working with the VIP at primary care centres in both urban and rural areas of Västerbotten County participated in the study.

### Data collection

Semistructured interviews were conducted by the first author (AS) between December 2024 and April 2025. The interview guide was developed by the research team to address the aims of the PRIMVIZA project and the study’s focus on cardiovascular risk communication in primary care (see the Supplementary File). The first interview was treated as a pilot interview. Following this interview, the research team reviewed the interview guide and found that it adequately captured the information relevant to the study aims. As no revisions were deemed necessary, the interview guide was used unchanged in subsequent interviews, and the pilot interview was included in the final analysis. Probing and follow-up questions were used throughout all interviews to encourage elaboration and clarification.

Two interviews took place in a private room at the participant’s workplace, while the remaining interviews were conducted via digital meetings (Microsoft Teams, Microsoft Corporation, Redmond, WA, USA), ensuring privacy for both the interviewer and the participant. No other persons were present during the interviews. The interviews were audio-recorded, and transcribed verbatim by the first author. No repeat interviews were conducted. Interview transcripts were not returned to participants for comment or correction.

### Data analysis

The interviews were analysed using inductive qualitative content analysis, following the approach described by Graneheim and Lundman [18] at a latent level of abstraction. The transcripts were read several times to gain an overall understanding of the material. Text segments relevant to the study’s aim were identified as meaning units, condensed while preserving their essential content, and interpreted into codes. Through comparisons of similarities and differences, the codes were organised into subcategories and categories.

Data saturation was assessed through ongoing analysis. After the tenth interview, no new aspects relevant to the study aim were identified, and this assessment was confirmed following analysis of the eleventh interview. Transcripts and codes were managed using MAXQDA (VERBI Software, Berlin, Germany). Participants did not provide feedback on the transcripts or the findings.

### Reflexivity and trustworthiness

The first author (AS), a primary care nurse with a doctoral degree, conducted all the interviews. We adopted an interpretivist epistemology, assuming that meaning is co-constructed through interactions between participants and researchers. To strengthen reflexivity, AS kept reflexive and analytic memos throughout data collection and analysis, documenting preunderstandings and reflections on potential influences on the research process.

Trustworthiness was enhanced through iterative discussions within a multidisciplinary research team with doctoral degrees in nursing, physiotherapy, and primary care medicine. Analytic interpretations and decisions were reviewed collaboratively until agreement was reached. Two members of the team were women and three were men. Careful documentation of analytic decisions and the use of illustrative quotations further supported transparency and confirmability.

There was no prior relationship between the interviewer and any participant. Before the interviews, participants were informed that the interviewer was a nurse researcher studying cardiovascular prevention in primary care. The interviewer’s professional background in preventive care was acknowledged and actively reflected upon throughout the research process.

## Results

### Sample characteristics

The sample consisted of 11 nurses working with the VIP health dialogues. Six participants were registered nurses with a postgraduate specialist nursing degree in primary care, and five were registered nurses without specialist training. Their professional experience as nurses ranged from 9 to 40 years (median 18.5 years), and their experience conducting VIP health dialogues ranged from 0.5 to 8 years (median 3 years). The participants were aged between 34 and 65 years (median 41 years), and two were men.

The interviews lasted between 38 and 60 min (median 49).

### Overview of findings

The analysis resulted in two main categories*: Meeting the person professionally while taking a step back* and *Continuously adapting approaches in self-management support.* These categories comprised four and five subcategories, respectively. Together, they formed the overarching theme *Shifting between leading and following*, which encapsulates the nurses’ overall experiences of communicating health risks and supporting discussions about lifestyle change within the context of VIP health dialogues (Table 1).

**Table 1:** Overview of the main theme, categories and subcategories

Main theme:	Shifting between leading and following	
Categories:	Meeting the person professionally while taking a step back	Continuously adapting approaches in self-management support
Subcategories:	Letting the person’s needs guide the conversation	Creating a casual “coffee break conversation”
	Understanding a person’s life situation and related resources	Presenting risks without inducing fear
	Exploring patients’ personal “why”	Using visual tools and metaphors
	Respecting different attitudes, even if they oppose one’s own	Basing arguments on guidelines and research
		Identifying simple, sustainable habits in daily life

### Main theme: shifting between leading and following

This overarching theme captures the nurses’ experiences of communicating cardiovascular risk and engaging in discussions about lifestyle-related issues during health dialogues. Central to these experiences was a continuous, conscious effort to attune the conversation to the individual in front of them, recognising their perspectives, life circumstances, and readiness to engage.

At the same time, the nurses described intentionally applying their professional strategies to support patients in exploring healthier behaviours in ways that felt meaningful and relevant. They portrayed a dynamic interplay between attentively holding the person’s values at the centre and providing explicit, evidence-based professional guidance, forming a foundation for dialogues that were described as aiming to encourage change while maintaining trust and autonomy.

### Meeting the person professionally while taking a step back

This category captures how nurses sought to balance professional guidance with respect for patients’ autonomy. They explained that throughout the health dialogue, they continually explored and acknowledged each individual’s unique situation, motivation, prior knowledge, and attitudes while deliberately avoiding dominating the conversation or imposing their own agenda.

### Letting the person’s needs guide the conversation

Nurses described how health dialogues could move beyond the initial focus on cardiovascular prevention, depending on what the patient chose to bring up. Topics such as mental health, stress, menopausal symptoms, and sleep problems were noted as emerging during these encounters. Nurses emphasised the importance of letting the patient guide the encounter, referring to what they described as the patient’s moment, and highlighted that the most meaningful dialogues were experienced when the patient spoke more than the nurse did. If a patient wished to focus on something other than cardiovascular risk, this was understood by the nurses as central to the person’s own experience of health. Such responsiveness was viewed as particularly important when psychological distress was present, which the nurses perceived as sometimes dominating the entire conversation.

In many cases, nurses reported beginning with the patient’s perceived health or general well-being rather than immediately addressing test results, a strategy they felt created space for reflection and feeling heard. Patients were described as frequently raising issues they had not previously had the opportunity to discuss in healthcare, making the dialogue an occasion to voice unspoken concerns. When mental health problems were prominent, nurses described prioritising these concerns before moving on to lifestyle-related risk factors and discussions about healthy behaviour.

“I always start by asking, ‘How are you?’ because I think that’s the most important question. I find that it allows you to get a bit deeper with the patient right away – it opens up a good conversation, and sometimes you end up spending a lot of time on mental well-being, on stress, and on all sorts of things. And then I think that maybe the other things are less important and can be dealt with later.” (Nurse 1)

Stress during the consultation itself was described by the nurses as a barrier, as tense or hurried patients were perceived as having limited ability to take in information, which led nurses to simplify, slow down, or postpone certain discussions. Language barriers were mentioned by the nurses as another challenge; interpreter-mediated dialogues were described as occurring, and nurses explained that nuances were often lost, making it harder to follow the patient’s lead and establish trust. The unpredictability of how the health dialogues unfolded was described as a characteristic feature of these encounters, underscoring the need to adapt to patients’ needs and priorities in the moment.

### Understanding a person’s life situation and related resources

Nurses emphasised the importance of understanding who the patient was and continuously taking this into account throughout the conversation. This included the person’s everyday circumstances, previous health experiences, self-perceived health, and existing health knowledge, as well as what specifically might motivate change.

According to the nurses, many patients had already reflected on their lifestyle, often prompted by the previsit questionnaire, with some having initiated changes or identified habits they wished to work on (e.g., physical activity or diet). Nurses noted that patients sometimes expressed that they already knew which lifestyle changes were recommended, which, in the nurses’ view, illustrated the questionnaire’s role as a reminder and catalyst for reflection. At the same time, knowledge levels were described as varying, even among patients who themselves worked in healthcare, and nurses therefore avoided making assumptions.

“We have nurses and doctors as patients. They don't have much knowledge about cholesterol; they become specialists in their areas. It might be a risk if you determine what the person works with too early.” (Nurse 3)

Nurses reported that patients’ self-rated health often revealed stress, fatigue, or emotional strain not captured by objective measures; such perceptions were described as influencing what to prioritise in the dialogue and what type of support to offer. They further highlighted that patients’ previous experiences and beliefs were crucial for tailoring the dialogue. Earlier attempts to change habits, such as dieting or increasing physical activity, were described as shaping patients’ confidence and motivation, where earlier successes could reinforce their sense of capability. Nurses also observed that misconceptions were common, for instance, believing that exercise had to last for hours to count. Exploring these factors was viewed as essential for adapting the dialogue to each person’s situation and readiness for change.

“Sometimes you think, ‘Wow, these are truly bad test results; how is this person actually feeling?’ And then they walk in feeling great, no health anxiety at all. You truly need a different approach for each person; no one is the same.” (Nurse 9)

Life circumstances were described as strongly influencing feasibility: major life events, demanding jobs, long commutes, personal health struggles, or illness in the family could disrupt habits and limit the capacity for change.

“We also have a life. Some may have started a lifestyle change, and then something happens; parents die, a sick child, or they get sick themselves, and they completely lose track.” (Nurse 3)

Nurses also reflected that age-related patterns appeared to matter. According to their accounts, around the age of 40, family responsibilities often took priority, limiting time for health and leading to simpler meals, whereas around the age of 60, physical limitations could affect activity. Addressing underlying issues such as stress or sleep problems was sometimes described as necessary before lifestyle changes were initiated. Overall, the nurses underscored the need for a personalised approach, continuously adapting the conversation to the patient’s unique situation.

### Exploring patients’ personal *why*

Nurses emphasised that identifying each patient’s personal motivation, their why, was important for supporting discussions about lifestyle change, noting that patients could be driven by very different reasons. Some were motivated by concrete goals, such as losing weight to feel lighter or fit into certain clothes, whereas others articulated performance-oriented goals, such as running a marathon. Nurses also noted that patients sometimes expressed broader health-related motivations, such as managing fatigue, sleeping better, or regaining lost physical capacity.

According to the nurses, it was not always obvious to patients what motivated them. In such cases, nurses described seeing it as important to explore this together and reflected that the health dialogue itself could help patients articulate their own why. They explained that uncovering these personal motivations was perceived as making change feel more meaningful and realistic, as illustrated by one nurse who recalled a woman linking her motivation to becoming a grandmother:

“She said, ‘My goal is to be able to carry my grandchild. To do that, I need to exercise.’ She had back issues. I thought that was so lovely.” (Nurse 8)

Nurses considered this individualised approach essential for tailoring support.

### Respecting different attitudes, even if they oppose one’s own

Nurses described encountering patients who expressed very different attitudes toward cardiovascular risk and lifestyle change, and emphasised the importance of respecting these attitudes, even when they contradicted general health recommendations. According to the nurses, many patients recognised areas for improvement in relation to their health and were perceived as responding positively when health-related benefits were explained. Some patients were described as expressing concern that change would require a complete overhaul but appeared relieved when they were told that small, gradual steps could be sufficient. In contrast, a few highly health-conscious patients were described as reacting with frustration when the results were not perfect.

At the same time, nurses reported encountering patients who resisted change. Some were described as openly prioritising perceived quality of life over long-term health, whereas others attributed risk to heredity or expressed acceptance of risk as unavoidable.

“I had a patient, I don’t know if he was 50 or 60, but he just said, ‘You know what, I’d rather die tomorrow than change any of my lifestyle habits. I’m fine with that.’” (Nurse 9)

Nurses also noted that some patients declined risk information altogether and attended the health dialogue only because they had been invited. When patients did not wish to receive further feedback, nurses described limiting the conversation to test results. Finally, nurses reflected that some patients did not perceive a need for change despite unfavourable results. These individuals were described as sometimes relying on information from social media or single studies, which led them to question or challenge standard advice.

“A lot of the challenge is, and it feels awful to say, that patients are well informed. However, it is skewed. They’re not informed in the areas where we are.” (Nurse 6)

### Continuously adapting approaches in self-management support

Nurses described relying on an extensive toolbox of strategies during the health dialogues to support discussions about lifestyle change. They emphasised that these strategies were never applied in a fixed way but were adapted flexibly to each patient and situation. Their intention was described as being to engage with patients’ perspectives, support understanding, and help make lifestyle changes feel relevant, realistic, and possible.

### Creating a casual “coffee break conversation”

Nurses described a clear intention to create a warm, informal, and respectful atmosphere in the health dialogues, often likened to a coffee break conversation. They emphasised avoiding a paternalistic stance and instead positioning themselves as supportive dialogue partners. In their view, this was perceived as helping patients feel at ease and allowing health topics to emerge naturally. To foster this atmosphere, nurses sometimes described sharing personal experiences during the health dialogue, such as struggles with healthy habits, body image, or balancing family life, which they felt helped to humanise them and reduce perceptions of judgement. According to the nurses, this kind of honesty, combined with empathy, was experienced as supporting patients in opening up and reflecting on their own behaviours.

In line with this relational approach, the nurses reported that motivational interviewing (MI) principles guided their work. They described aiming for dialogues in which patients spoke more than they did, and valued occasions when patients generated their own insights, identified realistic goals, and recognised the challenges of change.

“They often come in and say: ‘Oh well, now I’m here to get my verdict,’ and then I get the chance to prove them wrong by saying: ‘No, I’m not here to point fingers, we’re just having a dialogue.’ When they then acknowledge that and say at the end, ‘This turned out so much better than I thought it would. It was way nicer, and I feel motivated to get started,’ then I feel like I’ve succeeded.” (Nurse 8)

### Presenting risks without inducing fear

The nurses highlighted their professional and ethical responsibility to inform patients about health risks in accordance with scientific evidence, but they stressed that this needed to be done with sensitivity and awareness of each patient’s emotional state and readiness. They explained that their approach was adjusted depending on the person’s reaction; if someone became reluctant or explicitly declined further information, they often chose to hold back.

Fear-based communication was viewed as counterproductive, and a calm, factual tone was favoured in the health dialogue. In their experience, scare tactics risked triggering avoidance or disengagement, whereas respectful explanations combined with hope for improvement were more likely to encourage constructive engagement with one’s health.

“I don’t believe that shocking the patient achieves much. I rather think that they shut down in some way. They don’t take in anything you say.” (Nurse 4)

Maintaining patients’ autonomy and dignity was also described as a priority. To support this, the nurses reported deliberately avoiding judgemental language and trying to avoid provoking feelings of guilt or shame. This careful and respectful way of communicating was described as supporting patients in taking in risk information and helping to keep the dialogue open. They also reflected that some topics were more sensitive than others, noting that discussions about alcohol use and dietary habits were often perceived as delicate and could provoke defensiveness. In contrast, physical activity was generally viewed as less sensitive and easier to raise, sometimes serving as a smoother entry point for discussing lifestyle habits more broadly.

### Using visual tools and metaphors

Nurses reported relying on metaphors and visual aids as a way of supporting discussions about complex health information. They noted that everyday comparisons were perceived as working better than technical explanations. Another metaphor described lifestyle change as analogous to saving for retirement, where small, consistent efforts over time contribute to long-term benefits. Similarly, nurses used simple, relatable comparisons, such as comparing fat build-up in the blood to grease solidified in a frying pan. As one nurse explained:

“If they’re not receptive, I try using the example of a frying pan and bacon grease—that the blood vessels can become clogged, and that it gradually builds up over time if you don’t change your habits.” (Nurse 10)

Visual tools such as traffic-light charts developed by dietitians were described as being used to illustrate fat content in foods (green for healthy, yellow for moderate, red for foods to limit). According to the nurses, these simple images were perceived as helping patients grasp which choices were healthier. They added that brochures with clear colour coding were sometimes distributed, including to patients with normal values, as they were viewed as an accessible way to support cardiovascular prevention. Overall, visual aids were regarded as useful for making information more concrete, with charts, diagrams, and lists described as helping to simplify complex messages and as supporting patients’ openness and understanding.

### Basing arguments on guidelines and research

Nurses described feeling more secure when they based their advice on VIP guidelines and current research. By referring to established sources, such as recommendations from the National Food Agency or studies associated with the VIP and Umeå University, they explained that this allowed them to clarify that the guidance was grounded in scientific evidence rather than in their own personal views, which they perceived as reducing the risk that patients would view the advice as the nurse’s personal opinion or preference.

“I usually kind of say that it’s not me; it’s the guidelines. These are the recommendations we follow within the VIP. What I’m saying comes from those responsible for the programme. I’m not criticising you as a person; everything I say is based on the guidelines.” (Nurse 5)

Referring to objective test results, such as elevated blood pressure or LDL cholesterol, was described as another strategy to emphasise that the information was grounded in factual measurements and to provide patients with clear and concrete indications that lifestyle changes might be needed. When patients brought up alternative diets or single studies that conflicted with guidelines, nurses reported typically avoiding lengthy debates and instead returning to the information they were responsible for conveying. Some nurses also described encouraging patients to seek information from sources they considered reliable. Nurses emphasised that it was ultimately the patient’s decision whether to act on the advice, while they viewed their role as providing evidence-based support that patients could draw on in their own decision-making.

### Identifying simple, sustainable habits in daily life

Nurses emphasised that supporting a healthy lifestyle involved helping patients build on what already worked in their everyday lives while introducing adjustments that were realistic and sustainable. They described an approach centred on simplicity, gradual progress, and tailoring advice to everyday life, while deliberately avoiding restrictive or abrupt changes that were perceived as risking discouragement.

A recurring challenge, according to the nurses, was that many patients were perceived as believing they had to change everything at once, which was described as often leading to relapses. To address this, nurses reported promoting step-by-step change and working with patients to identify small, achievable goals, such as standing once an hour, taking a short walk, or adding a vegetable to a meal. They noted that such low-barrier changes were sometimes experienced as opening up possibilities for broader adjustments; for example, nurses described instances where patients who quit smoking appeared to regain stamina, became more active, and expressed motivation to further work on their health.

Nurses also highlighted the value of enjoyment and intrinsic motivation. Open questions such as “What kind of activity would work for you?” were described as a way of ensuring that suggestions were relevant and feasible. When several lifestyle areas required attention, nurses described breaking these down and prioritising them together with patients to avoid overwhelming them.

Addressing everyday barriers to a healthier lifestyle was viewed as equally important. Nurses shared examples of practical solutions adapted to daily routines, such as taking walks during children’s sports practices, preparing extra food for lunches, or modifying meals to suit family preferences. They reflected that when changes were simple and nondisruptive, patients appeared more willing to try to maintain them. Nurses recalled encounters with patients who initially seemed sceptical but later returned to the clinic or were observed in the community engaging in physical activity, experiences that reinforced nurses’ perceptions of patients’ capacity to work towards meaningful change.

*“Almost everyone says, ‘I’ve tried but I haven’t succeeded.’ I usually tell them not to set goals that are too big but to start with short-term ones. It’s not about doing a workout but maybe taking a 5- or 15-minute walk during lunch. I believe it’s about not changing everything at once but making small adjustments; otherwise, it will never work.”*

(Nurse 2)

## Discussion

This study explored primary care nurses’ experiences of cardiovascular risk communication and lifestyle counselling within structured preventive health dialogues. The findings indicate that nurses experienced risk communication as a relational and adaptive process, characterised by continuous shifts between leading and following the patient. Rather than applying fixed communication strategies, nurses described actively attuning their dialogue to patients’ readiness, life circumstances, emotional states, and expressed priorities, while simultaneously drawing on professional knowledge, clinical guidelines, and structured tools. In doing so, this study provides contemporary qualitative insight into how lifestyle risk communication is enacted in routine preventive primary care practice within an established population-based programme. By focusing on nurses’ experiential accounts, the study extends existing literature that has largely examined barriers or communication techniques and instead highlights the skilled interactional work underpinning preventive primary care nursing.

International research has described lifestyle risk communication in primary care as an interactional and relational process embedded in ongoing nurse–patient relationships, rather than as a one-way transfer of information [24, 25]. The present findings align with this literature by illustrating how nurses emphasised rapport, trust, and patient-led dialogue when discussing cardiovascular risk. At the same time, this study extends previous work by offering a more fine-grained account of how nurses continuously navigate the tension between professional responsibility and respect for patient autonomy within the same consultation. The overarching theme, Shifting between leading and following, captures this dynamic process and adds empirical depth to earlier conceptual descriptions of interactional risk communication.

This pattern can also be understood in relation to the continuum of communication styles described in motivational interviewing, ranging from directing to following, with a guiding approach positioned in between. Within this framework, guiding involves balancing the provision of information with responsiveness to the individual’s perspectives and motivations. The nurses’ descriptions in this study reflect such a dynamic practice, where communication is adapted to the situation and the individual, rather than being strictly directive or non-directive. In addition, the emphasis on exploring patients’ own motivations and allowing the dialogue to be shaped by the individual reflects key elements of motivational interviewing, suggesting that these communication practices align with its core principles in the context of preventive cardiovascular dialogues [41].

Observational studies in primary care have shown that lifestyle counselling is often delivered in relatively generic terms and that motivational interviewing principles are applied inconsistently across consultations and professionals [43, 44]. In contrast, nurses in the present study described deliberate and ongoing individualisation of their communication. Variation in counselling approaches did not appear to reflect a lack of structure or competence but rather an intentional response to the complexity of patients’ situations, including mental health concerns, competing life demands, and differing levels of motivation.

Previous research on lifestyle counselling has primarily focused on barriers such as time constraints, organisational conditions, and perceived patient motivation [4, 7, 26, 57]. Similar challenges were also described by nurses in the present study, including limited opportunities for follow-up, staff shortages, and the prioritisation of acute issues, which at times made preventive dialogues feel like a “one-shot opportunity” despite the gradual nature of behaviour change. These constraints mirror broader systemic challenges in primary care prevention, such as reimbursement structures, limited interdisciplinary support, and competing clinical priorities [3, 28, 31]. However, rather than positioning these barriers solely as limiting factors, the present findings illuminate how nurses described working within such constraints by prioritising issues, adjusting pacing, and selectively introducing risk information based on patients’ emotional readiness and expressed concerns. This highlights a core tension in preventive practice: while standardised tools and guidelines provide necessary structure, meaningful and sustainable behaviour change requires flexibility and room for person-centred, responsive communication [3].

Nurses in this study described how communication was continuously adjusted in real time based on patients’ cues and responses, reflecting a relational epistemology in which knowledge is cocreated rather than delivered unilaterally. These accounts align with previous research showing that risk information alone rarely leads to behaviour change [2, 6] and underscore that effective prevention depends on trust-building and coconstructed meaning [49]. Allowing dialogues to shift focus toward stress, sleep, or mental health when such issues were prerequisites for engagement reflects a communicative balancing also described in earlier qualitative research on preventive health dialogues [23]. Since that study, preventive practice has evolved through increased emphasis on person-centred care, expanded use of structured guidelines, and pedagogical and visual tools, developments that appear to have added layers of complexity rather than replacing relational work.

Within this adaptive communicative practice, nurses described tailoring strategies to each patient’s knowledge, resources, and motivation, reflecting principles of person-centred care [8] and motivational work in which motivation is understood as emerging through interaction rather than as a fixed trait [12]. Taken together, this pattern reflects a broader shift in preventive care from compliance-oriented models toward concordance-based approaches, where shared understanding and negotiated meaning are central [51]. Such practices highlight the limitations of one-size-fits-all prevention models and the need to balance standardised protocols with relational responsiveness [34].

Further, nurses described how the importance of pacing became particularly evident when multiple risk factors were present. They highlighted the emotional labour involved in balancing honesty with hope, especially when test results were unfavourable or when patients appeared unmotivated. Nurses described deliberately slowing down, prioritising, or postponing certain messages to avoid overwhelming patients. This aligns with previous research showing that excessive or poorly timed risk communication can provoke anxiety, avoidance, or disengagement [34]. Similar patterns were described by Hörnsten et al. [23], who showed that nurses in preventive health dialogues sought to inspire confidence rather than fear—an approach echoed in the present findings, where fear-based communication was described as risking disengagement rather than supporting change.

Several strategies described by nurses correspond to established behaviour change techniques, such as goal setting, action planning, and prompts or cues [38]. Encouraging small, feasible steps, such as short walks or minor dietary adjustments, reflects an understanding of behaviour change as incremental and situated within everyday life. This emphasis on gradual change is consistent with the Common-Sense Model of Self-Regulation, which suggests that engagement with risk information depends on its fit with individuals’ beliefs, emotions, and coping capacity [32].

Communication strategies consistent with motivational interviewing were also evident in the nurses’ accounts. They described using open questions and reflective strategies to help patients articulate their personal “why” for change and to strengthen intrinsic motivation [40]. Their intentional use of a warm, informal “coffee-chat” tone likewise supported openness and trust, while helping to preserve patients’ dignity by avoiding blame. Similar communication patterns have been reported in earlier studies, where nonthreatening and relational approaches have been shown to facilitate engagement and sustain involvement in preventive health dialogues [14, 27].

To support comprehension, the nurses in this study used everyday metaphors and visual aids, traffic-light tables, and star profiles to translate abstract risk into concrete and relatable terms. These findings align closely with prior research showing that tailored and clearly framed explanations benefit patients across health literacy levels, supporting understanding for those with lower familiarity with health information while still adding clarity for those with more knowledge [11, 21, 49]. Related evidence further suggests that personalised visualisation tools, including heart-age graphics and carotid ultrasound images, can enhance risk perception and motivation by making risk more concrete and emotionally salient [46]. This connects to broader development work within PRIMVIZA, where the implementation of ultrasound-based visualisation in VIP dialogues is being examined at an organisational and pedagogical level.

Importantly, these communicative strategies intersect with equity concerns. The nurses reported that language barriers, cultural differences, and differences in comprehension tended to complicate communication, echoing prior research on the need for culturally adapted strategies. Without tailored resources, there is a risk of inverse care, where individuals with the greatest needs (e.g., low health literacy, complex life situations, diverse cultural backgrounds) may receive the least usable support, potentially exacerbating health inequities [13].

Taken together, these findings suggest that cardiovascular prevention in primary care is a communicative and relational task, not only a biomedical task. By showing how nurses balance structured guidance with person-centred dialogue, this study underscores the importance of organisational conditions that enable such work and highlights the potential value of visualisation tools. Our findings therefore reinforce the rationale for designing preventive interventions in primary care as interactive, relational processes rather than one-way information delivery.

### Strengths and limitations

The participating nurses varied in age and professional experience, which strengthens the credibility and potential transferability of the findings [19]. The gender distribution among participants (nine women and two men) reflects the composition of the primary care nursing workforce in Sweden, where the profession is predominantly female. Although this limits gender diversity, it enhances contextual representativeness.

The interviewer’s background as a primary care nurse may have influenced the interviews. Interviewing colleagues with a shared professional background can offer advantages, such as deeper understanding and the ability to ask relevant follow-up questions [37], but it also entails a risk that shared assumptions remain unexamined [36]. To address this, reflexivity was emphasised throughout the research process, with regular discussions within the research team to reflect on assumptions and potential biases [9].

Trustworthiness was further supported through purposive sampling, team-based analysis, and the use of established qualitative methods [18]. Credibility was enhanced through triangulation and the inclusion of illustrative quotations. Dependability and confirmability were strengthened by transparent reporting and grounding interpretations in the data [33]. Adherence to the COREQ checklist further contributed to methodological rigour and transparency.

Nevertheless, several limitations should be acknowledged. Participation may have been more attractive to nurses with a particular interest in prevention or strong views on cardiovascular counselling, introducing potential selection bias. In addition, all interviews were conducted within the VIP in northern Sweden, which may limit transferability to other healthcare systems or preventive settings. As a qualitative study, this research does not aim to assess the effectiveness of specific communication strategies or outcomes, but to explore nurses’ experiences and perceived practices related to cardiovascular risk communication. However, the detailed description of the study context, participants, and analytic process supports assessment of relevance to similar primary care settings.

## Conclusions

This study illustrates how nurses in primary care experience and navigate cardiovascular risk communication and lifestyle counselling during preventive health dialogues. Nurses described a continuous balancing between professional guidance and responsiveness to each person’s situation, emotions, and readiness for change. Through this relational and adaptive approach, risk communication was described as shaped to make information meaningful and manageable in everyday life.

The findings suggest that preventive communication in primary care requires organisational conditions that allow time, continuity, and relational engagement, enabling dialogues that extend beyond information delivery alone. In the context of PRIMVIZA, the findings can inform the ongoing development of preventive health dialogues within the VIP by highlighting how nurses integrate structured tools with person-centred communication in practice. Future research should explore how visual and pedagogical tools can be sustainably integrated into routine primary care and examine whether and how such approaches are associated with more equitable cardiovascular prevention. Within the PRIMVIZA project, this includes investigating how visualisation, in combination with person-centred risk communication as part of routine preventive dialogues, may support patients’ understanding, motivation, and lifestyle behaviours.

### Implications for practice

- Preventive consultations may benefit from being approached as dynamic, dialogical processes, in which clinicians actively shift between leading and following the patient, balancing professional guidance with responsiveness to individual needs, preferences, and readiness for change. Structured tools may support this process but do not replace relational adaptation and motivational work.
- Visualisation tools may be particularly helpful when they are introduced sensitively and linked to the patient’s own goals, values, and everyday circumstances, suggesting that such tools could complement existing communication strategies rather than function as stand-alone solutions.
- Preventive work may benefit from organisational conditions that allow time, continuity, and opportunities for follow-up, especially for patients with low health literacy, complex life situations, or multiple risk factors.

## Supplementary Information

Supplementary Material 1.

Supplementary Material 2.

## Data Availability

Owing to Swedish legislation on research confidentiality, full interview transcripts cannot be made publicly available. Anonymised excerpts relevant to the findings are available from the corresponding author upon reasonable request.

## Abbreviations

CVD: Cardiovascular Disease
MI: Motivational Interviewing
PRIMVIZA: PRIMary care prevention of cardiovascular disease using VIsualiZAtion
VIP: Västerbotten Intervention Programme
VIPVIZA: Västerbotten Intervention Programme-VIsualiZAtion of asymptomatic atherosclerotic disease trial
WHO: World Health Organisation
