# Supplementary material for: Nurses’ experiences with cardiovascular risk communication and lifestyle counselling during preventive dialogues in primary care: a qualitative interview study
Source: BMC Prim Care. 2026 Jun 4;27:225. doi: 10.1186/s12875-026-03401-7 (PMC13242669; doi:10.1186/s12875-026-03401-7)
Supplement: Supplementary file 2 — Supplementary Material 2. [file 12875_2026_3401_MOESM2_ESM.docx]

**Interview guide**

- Can you describe some challenges you have encountered when communicating health risks to patients?
- How do you perceive that patients usually react when needs for changes in their lifestyle habits are expressed, related to cardiovascular disease or other conditions, during the VIP health dialogue?
- Based on your experience, what types of communication or information tend to work well in helping patients change their lifestyle habits? What works less well? Please give examples.
- What factors among healthcare professionals or within the healthcare system, and what factors among patients, do you think facilitate or hinder patients in making lifestyle changes?
- What do you believe are the most important factors influencing patients’ motivation to change their lifestyle habits or to follow medical recommendations?
- How do you perceive that patients integrate information about their health risks into their everyday lives?
